# Page Kidney: An Unusual Complication of a Renal Transplant Biopsy

**DOI:** 10.1155/2018/8768549

**Published:** 2018-04-24

**Authors:** Jacob D. McFadden, Jason S. Hawksworth

**Affiliations:** Department of Transplant Surgery and Department of Urology, Walter Reed National Military Medical Center, 8901 Wisconsin Avenue, Bethesda, MD, USA

## Abstract

Page kidney, a rare phenomenon whereby external compression of renal parenchyma can induce hypertension, can be caused by subcapsular hematoma following renal transplant biopsy. Surgical intervention is often warranted to salvage the transplant kidney. This is a case report of a patient with acute T-cell-mediated rejection and no other risk factors for postprocedural bleeding that developed Page kidney. The patient had no signs or symptoms for >24 hours from the time of biopsy, underscoring the need for awareness of this rare but potentially catastrophic complication of renal transplant biopsies.

## 1. Introduction

Page kidney is a phenomenon first described by Irving Page in 1939. His experiments wrapping canine kidneys in cellophane were notable for the induction of hypertension and a perinephric inflammatory response in the test subjects [1]. The pathophysiology of Page kidney is external compression of renal parenchyma causing hypoperfusion of the kidney; this triggers activation of the renin-angiotensin-aldosterone (RAAS) axis and results in systemic hypertension. The phenomenon was observed sparingly after its initial description, seen primarily in football and trauma-related cases; more recently, etiologies as varied as cysts, aneurysms, retroperitoneal tumors, and lymphoceles have been reported [2–4]. Presentation typically includes a drop in hemoglobin with new hypertension—a recent analysis reported an average blood pressure of 177/95 mmHg [2]. When bilateral kidneys, or a solitary or transplant kidney is involved, worsening renal function will coincide. Diagnosis is typically confirmed radiographically, via ultrasound or computed tomography. Treatment options include medical management (including antihypertensives that affect the RAAS axis), percutaneous drainage of para- and perirenal fluid collections, surgical drainage of hematomas via capsulotomy/capsulectomy, and nephrectomies [3–6]. There is a very low incidence of transplant kidney biopsy complications overall. A recent large single-center retrospective review noted significant complications in 1.9% of cases, with interventional radiology or surgical interventions required in only 0.7% of cases [7]. Biopsy-related Page kidney was more frequent during a recent review of cases reported since 1991 [2]. With an increasing population of renal transplant recipients, an increased frequency of renal transplant biopsies may partially account for the apparent rise in associated Page kidney incidence. Nonetheless, despite the infrequency of this condition, the significant consequences of untreated Page kidney make it a worthwhile consideration in the care of renal transplant patients that have undergone recent biopsy.

## 2. Case Report

The patient is a 63-year-old male with a history of ESRD of unclear etiology, who underwent an uncomplicated cadaveric renal transplant with a creatinine nadir of 1.3 mg/dl. Approximately 6 months postop his creatinine level demonstrated an increase (1.78 mg/dl) and he developed new proteinuria in the setting of medical noncompliance. He underwent workup for allograft dysfunction, which included a normal renal transplant ultrasound; negative testing for DSA, BK virus, and CMV; review of blood tacrolimus levels; and maintenance of blood pressure control.

He underwent a standard ultrasound-guided renal transplant biopsy under sedation with the interventional radiology team, with two cores obtained from the lower pole of the transplant kidney. No complications were observed during the procedural imaging. He was not on any anticoagulants nor antiplatelet medications at the time of the procedure. Per standard practice, the patient was monitored for four hours after the procedure, had no pain and no significant bleeding on his dressing, and had normal vital signs. He was voiding normal yellow urine prior to discharge that afternoon.

The following day, his biopsy results confirmed acute T-cell-mediated rejection (Banff Grade IIB). He was contacted and admitted to the hospital for planned thymoglobulin infusion that evening. On arrival, his vitals were normal with a BP of 133/88 mmHg; however, over the course of the evening, his blood pressure rose to a max of 170/102 mmHg and he developed nausea. His admission labs returned with a notable creatinine rise from 1.31 to 4.46 mg/dl. His hemoglobin was stable at 12.1 g/dl with normal platelets and coagulation profile. He denied pain or any issues with urinating, including hematuria or low urine volume, since his biopsy. His exam was benign—no back, flank, or abdominal tenderness.

**Figure 1:**
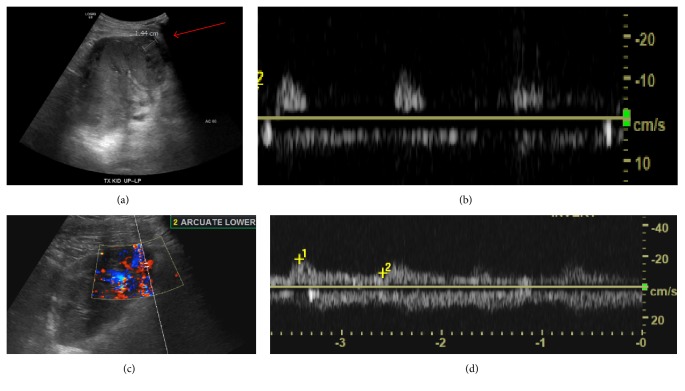
(a) Preprocedure renal transplant ultrasound demonstrating subcapsular hematoma (arrow). (b) Arcuate waveforms from the inferior pole with RI = 1.0 prior to operative decompression. (c) Renal transplant ultrasound after capsulotomy and clot evacuation, demonstrating restoration of inferior pole perfusion with (d) normalization of arcuate artery RI to 0.7.

The precipitous uptick in his creatinine level combined with his hypertension and mild nausea raised concerns for complication from his recent renal biopsy. A renal transplant ultrasound was obtained, which demonstrated a subcapsular hematoma (Figure 1(a)) in addition to decreased color Doppler flow at the inferior pole of the transplant kidney. These findings prompted urgent operative intervention. The patient underwent an exploratory laparotomy which revealed subcapsular hematoma spanning the entire allograft with circumferential compression. This was treated surgically with generous capsulotomy, releasing approximately 500 cc of clotted blood. Meticulous inspection of the allograft failed to identify a primary bleeding source, but hemostasis was achieved with a combination of electrocautery and topical hemostatics* (Surgicel Original Hemostat, Surgicel Fibrillar Hemostat)*. The allograft appeared pink and well perfused at the conclusion of the procedure. An intraoperative ultrasound (Figure 1(b)) confirmed interval improvement in color flow at the superior pole and periphery of the kidney as compared to the preoperative exam. A Jackson-Pratt drain was placed prior to closing the abdomen.

**Figure 2:**
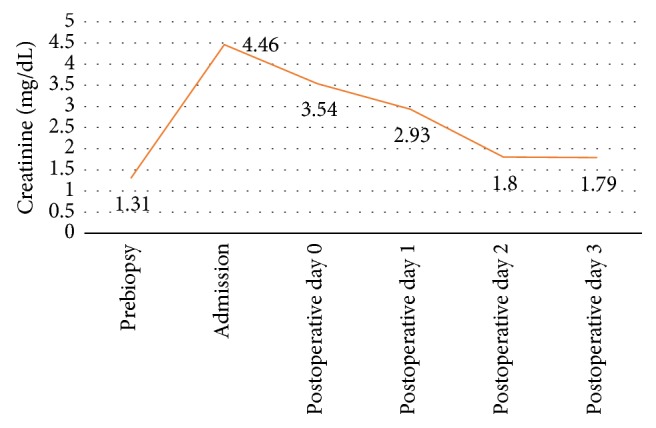
Creatinine trend in perioperative setting.

A repeat transplant kidney ultrasound was performed on postoperative day 1, again documenting improved vascular flow in the transplant kidney with normal resistive indices (0.6–0.7) and patent venous drainage; no perinephric fluid nor hydronephrosis was identified. The patient recovered well and showed immediate improvement in his creatinine (Figure 2); he was discharged on postoperative day 3 after removal of his drain and completion of three total doses of IV thymoglobulin for his rejection. At the time of discharge, his vitals were stable with blood pressure ranges of 112–127/70–88 mmHg. His creatinine was 1.79 mg/dl and his CBC was normal. His wounds healed well, his blood pressure returned to baseline, and his creatinine level at clinic follow-up on June 21, 2017, was 1.30 mg/dl.

## 3. Discussion

The majority of renal transplant biopsy complications will present within 24 hours of the procedure [7], though complications that present after this time period are still able to be treated. Page kidney, a phenomenon whereby external compression of renal parenchyma induces hypertension and decreased renal function in renal transplant patients, is an unusual complication of renal transplant biopsies despite the high number of renal transplant biopsies performed. Others have reported chief signs and symptoms of Page kidney including acute pain over the graft, alteration in blood pressure control, reduction of urine output, Doppler US evidence of subcapsular hematoma, and elevated resistive indices, as pathognomonic of this phenomenon [3]. This case report of Page kidney in a patient with acute cell-mediated rejection and no other risk factors or medications for postprocedural bleeding [8], presenting with minimal symptoms and significant decline in renal transplant function, highlights the need for vigilance in the postbiopsy period to ensure graft viability. A similar case of Page kidney in a patient with acute humoral rejection, reported by Chung et al. in 2008, resulted in the patient becoming dialysis-dependent despite surgical decompression [3]. In a comparison of traumatic and nontraumatic causes of Page kidney, nontraumatic cases (including biopsy of native, nontransplant kidneys) had a higher rate of nephrectomy [9]. A high degree of suspicion for the condition and early utilization of imaging modalities, including fast and low-risk renal transplant ultrasounds, can confirm the diagnosis. While emerging noninvasive technologies [10] for rejection in the transplant kidney may reduce the need for invasive testing in the future, postprocedure monitoring should currently be considered paramount in these patients to prevent renal transplant failure.
